# Men's Perspective Toward Menstruation: A Cross-Sectional Study

**DOI:** 10.7759/cureus.90701

**Published:** 2025-08-21

**Authors:** Sri Varsha, Parthiban Vasudevan, Kavya Palanisamy

**Affiliations:** 1 Community Medicine, Sree Balaji Medical College and Hospital, Chennai, IND; 2 Surgery, Apollo First Med Hospitals, Chennai, IND; 3 Community Medicine, Karpagam Faculty of Medical Sciences and Research, Coimbatore, IND

**Keywords:** gender role, male respondents, menstrual attitude, menstruation stigma, reproductive health

## Abstract

Introduction

Despite increasing attention toward menstrual health in India, male perspectives remain largely neglected. Most menstrual health studies are female-centric, overlooking men’s roles in perpetuating or challenging menstrual stigma. This study explores the attitudes of Indian male university students toward menstruation using a culturally adapted Menstrual Attitude Questionnaire.

Methods

This cross-sectional study was conducted among 300 male students aged 18-45 years from various academic disciplines in a deemed-to-be university in the Chengalpattu district, Tamil Nadu, India. Participants were selected via stratified random sampling. A prevalidated, semi-structured questionnaire was administered, comprising sociodemographic items and the MAQ-M instrument. Statistical analyses, including descriptive statistics, independent t tests, and multivariate analysis of variance (MANOVA), were conducted using IBM SPSS Statistics for Windows, Version 26 (Released 2018; IBM Corp., Armonk, New York), with a significance level set at p < 0.05.

Results

Participants demonstrated high mean scores in Natural Perception (5.97 ± 0.69) and Empathy/Supportive Attitudes (5.82 ± 0.75), indicating positive and empathetic views toward menstruation. However, Debilitating Attitudes (5.57 ± 0.82) and Cultural Stigma Beliefs (4.53 ± 0.79) remained prevalent, reflecting ongoing misconceptions. Statistically significant associations were observed between menstrual attitudes and variables such as academic discipline (p = 0.002), educational level (p < 0.001), and residential status (p = 0.015). MANOVA confirmed the multivariate effect of education and department on overall menstrual attitudes.

Conclusion

This study reveals a dichotomy in male students’ menstrual attitudes, wherein empathy and awareness coexist with entrenched cultural stigma. Health-related education and postgraduate academic standing were significantly associated with more progressive views. The findings highlight the need for inclusive menstrual health education across all academic streams.

## Introduction

In India, most studies on menstruation include only women as participants, thus making it a women’s topic. This exclusion of men fails to recognize menstruation as a social epidemiological entity with existing gender inequalities [1]. Menstruation is still a taboo subject, and an important element in this taboo process is the negative attitudes expressed by young men toward menstruation and the emotional impact this can have [2]. Within lower socioeconomic groups, menstruation remains a subject avoided in public discourse, while in middle- and upper-income strata it is typically addressed in hushed tones or through euphemistic expressions. Mothers, irrespective of education level, tend to be very conservative when talking about menstruation with their boys [1]. Indian men, including educated professionals, are not only uncomfortable talking about menstruation but also find it unmanly to do so [3]. Male opinions on menstruation have not received much attention from scholars, but a thorough grasp of the topic in society at large could protect future generations from men’s overall lack of interest and women’s needless shame [4].

Boys in India mostly learn about menstruation and puberty from unofficial sources, overheard conversations, and cultural customs. The social, family, school, and peer contexts have the greatest influence on late-adolescent sexuality [5]. Due to rigid gender standards, teenage boys may isolate themselves from the physiological and social experiences of girls and remain ignorant of the subject, making menstruation a gendered experience. An immediate impact of their unawareness could be on girls’ self-esteem and mobility, as they face teasing about menstruation at this sensitive age [6]. Moreover, among other social variables, rigid gender roles, constrictive gender norms, and the gendered character of menstruation may have a detrimental effect on girls’ reproductive health decisions and exacerbate the consequences of sexual and reproductive risk factors on their current and future health [6,7].

While many studies aim to understand and improve girls’ menstrual hygiene management, boys’ attitudes and knowledge of the topic remain underexplored, limiting their inclusion as agents of positive change. Boys who grow up with a lack of knowledge about women’s reproductive health and the menstrual cycle may be more susceptible to unintended pregnancies, pregnancy complications, and sexually transmitted diseases (STDs) [8,9]. While most awareness programs focus on women, men’s attitudes significantly influence menstrual health outcomes, access, and stigma. However, limited research exists on men’s understanding and perceptions of menstruation, particularly among educated youth. Guided by the Social Determinants of Health framework and Gender and Power Theory, this study views male attitudes toward menstruation as products of social learning, cultural norms, and access to accurate information, shaped by factors such as education, peer influence, and living environment [10].

Accordingly, this study aimed to quantitatively assess Indian male university students’ attitudes toward menstruation across defined attitude domains, compare differences by academic discipline and education level, and identify sociodemographic predictors of supportive behavior.

This article was previously presented as a poster at the Medzantica’22 National Level event held on October 7 at Sri Lakshmi Narayana Institute of Medical Sciences, Puducherry, India.

## Materials and methods

Material and methodology

This study was a cross-sectional design aimed at evaluating men’s attitudes toward menstruation. To establish an appropriate sample size, Cochran’s formula was utilized [10]. The prevalence (p) was sourced from Rana et al. (2022), who found that 41.4% of men exhibited poor knowledge regarding menstruation in their cross-sectional survey of male populations in central India [11]. The margin of error was set at 5%. Consequently, the minimum sample size calculated was 373. Due to a non-response rate of approximately 19.6%, attributed to the sensitive nature of the topic, the final sample consisted of 300 male university students. Stratified random sampling was used to ensure representation from MBBS, nursing, dental, law, architecture, and allied health sciences. The sampling frame was prepared from departmental enrollment lists obtained from the academic registry. Each department served as a stratum, and the sample from each was allocated proportionally to its share of the total university enrollment: MBBS (n = 120), Nursing (n = 45), Dental (n = 30), Allied Health Sciences (n = 25), Law (n = 60), and Architecture (n = 20). Within each stratum, participants were randomly selected using computer-generated numbers without replacement, ensuring adequate representation of all subgroups. The study was conducted in a deemed-to-be university in Chengalpattu District, Tamil Nadu, India, with ethical clearance. The study duration was from September 2022 to February 2024. Male participants aged 18-45 years from academic backgrounds were included. The exclusion criteria were participants with notable hearing or visual impairments that would hinder their ability to complete the survey, as well as students who had previously participated in studies regarding menstrual attitudes or similar subjects, to prevent bias or contamination of responses.

The data were collected using the Menstrual Attitude Questionnaire - Indian Version (2024), which had been validated [12]. The questionnaire comprised two sections. Section I included questions pertaining to the sociodemographic profile, while Section II featured the Menstrual Attitude Questionnaire - Indian Version (2024). This specific questionnaire was designed to assess men’s views on menstruation and their behaviors toward individuals who menstruate. The Menstrual Attitude Questionnaire is divided into seven subscales: Menstruation as a Debilitating Event, Bothersome Event, Natural Event, Anticipation and Prediction, Denial of Any Effect, Cultural Belief and Stigma, and Empathy and Supportive Behavior. Each subscale was evaluated using a seven-point Likert scale. The Menstrual Attitude Questionnaire - Indian Version (2024) has shown high internal consistency (Cronbach’s alpha > 0.7), confirming its reliability for this study. After obtaining informed consent, self-administered questionnaires were distributed in small groups under the supervision of trained research assistants, and completed forms were immediately reviewed for completeness to minimize missing data. Once collected, the data were entered into MS Excel sheets (Microsoft Corporation, Redmond, Washington), and analysis was performed using IBM SPSS Statistics for Windows, Version 26 (Released 2018; IBM Corp., Armonk, New York). The analysis included descriptive statistics to summarize the mean and standard deviation for each of the seven subscales and independent t-tests to evaluate the impact of departmental background and academic level on participants’ attitudes toward menstruation. The t-test compared the mean scores on the subscales for these subgroups to identify any statistically significant differences, and multiple linear regression was conducted to explore the predictors of supportive behavior concerning menstruation. Factors including age, department, educational attainment, and understanding of menstruation were incorporated into the regression model. All statistical analyses were conducted at a significance threshold of 0.05.

Ethical considerations

Regarding ethical considerations, approval from the Institutional Human Ethics Committee was obtained before starting the study (IHEC-I/1229/22). All participants were informed about the purpose of the study, its benefits, the procedure, and the confidentiality of the research in both their local language and English. Written informed consent was obtained from those willing to participate. The study adhered to ethical guidelines for research involving human participants. It was reviewed and approved by the university’s Research Ethics Board to ensure compliance with standards for confidentiality, informed consent, and voluntary participation.

## Results

A total of 300 Indian male participants, aged 18 to 45 years, were surveyed. The distribution of key sociodemographic variables is presented in Table 1.

**Table 1:** Sociodemographic characteristics of participants (n = 300)

Variable	Category	Frequency (%)
Age	18–30 years	250 (83.4%)
	31–45 years	50 (16.6%)
Department	Health Sciences (MBBS, Nursing, Dental)	195 (65.0%)
	Non-health (Law, Architecture)	105 (35.0%)
Education level	Undergraduate	230 (76.7%)
	Postgraduate	70 (23.3%)
Residence	On-campus	120 (40.0%)
	Off-campus	180 (60.0%)

Table 1 shows that the study population primarily consisted of young adult males (83.4% aged 18-30 years), with 16.6% aged above 30. The majority were enrolled in health-related academic programs (65.0%), including MBBS, Nursing, and Dental, while the remaining 35.0% pursued non-health disciplines such as Law and Architecture. Most participants were undergraduate students (76.7%), and a larger proportion resided off campus (60.0%), which may reflect greater exposure to the external community or comparatively limited access to institutional health resources. These variations are important for interpreting how factors such as age, education level, discipline, and residential environment influence attitudes captured in the Menstrual Attitude Questionnaire - Indian Version (2024) subscales.

Table 2 summarizes the mean scores and standard deviations for each subscale of the Menstrual Attitude Questionnaire - Indian Version (2024), measured on a 7-point Likert scale (1 = Strongly Disagree, 7 = Strongly Agree).

**Table 2:** Distribution of scores across the Menstrual Attitude Questionnaire - Indian Version (2024) (n = 300)

Subscale	Mean ± SD	Min–Max
Debilitating attitudes	5.57 ± 0.82	3.41–7.00
Bothersome feelings	3.99 ± 0.78	1.32–6.16
Natural perception	5.97 ± 0.69	3.89–7.00
Anticipation awareness	5.05 ± 0.77	2.90–7.00
Denial of effects	3.04 ± 0.83	1.00–5.80
Cultural stigma beliefs	4.53 ± 0.79	2.29–6.55
Empathy/supportive	5.82 ± 0.75	3.70–7.00

According to Table 2, the Natural Perception subscale recorded the highest mean score (5.97 ± 0.69), indicating that most participants strongly acknowledged menstruation as a natural biological process. The Empathy/Supportive subscale (5.82 ± 0.75) also demonstrated high levels of emotional support and willingness to assist menstruating individuals. In contrast, the Denial of Effects subscale yielded the lowest mean score (3.04 ± 0.83), suggesting that participants largely recognized the physical and emotional impact of menstruation rather than trivializing it. The Debilitating Attitudes subscale scored relatively high (5.57 ± 0.82), pointing to a persistent belief among many men that menstruation impairs women’s functionality. Bothersome Feelings (3.99 ± 0.78) and Cultural Stigma Beliefs (4.53 ± 0.79) showed moderate scores, reflecting a mix of openness alongside residual discomfort and traditional beliefs. Overall, the descriptive data reveal a contradictory and evolving pattern: while participants expressed empathy and acknowledged menstruation as natural, some misconceptions and cultural biases remained.

According to Table 3, independent samples t-tests were conducted to evaluate whether menstrual attitudes varied across sociodemographic variables, including age group, department, educational level, and residence. For Anticipation Awareness, scores were significantly higher among non-health students compared with health sciences students (5.26 ± 0.66 vs 5.09 ± 0.69; t = -2.08, p = 0.038) and among postgraduates compared with undergraduates (5.38 ± 0.70 vs 5.09 ± 0.67; t = -2.91, p = 0.004). Bothersome Feelings were also significantly higher among postgraduates than undergraduates (4.37 ± 0.83 vs 4.07 ± 0.68; t = -2.95, p = 0.003). For Cultural Stigma Beliefs, non-health students scored significantly higher compared with health sciences students (4.82 ± 0.82 vs 4.53 ± 0.76; t = -4.26, p = 0.001). Natural Perception scores were significantly higher among on-campus residents compared with off-campus residents (5.98 ± 0.62 vs 5.81 ± 0.56; t = -2.45, p = 0.015). Empathy/Supportive attitudes were significantly higher among postgraduates compared with undergraduates (6.06 ± 0.57 vs 5.72 ± 0.70; t = -3.45, p = 0.001). No statistically significant differences were observed for Debilitating Attitudes or Denial of Effects across any sociodemographic variables (p > 0.05).

**Table 3:** Association between sociodemographic characteristics and menstrual attitude subscales (n = 300) *Statistically significant at p < 0.05. Independent samples t-tests were applied to compare mean scores across sociodemographic groups.

Subscale	Factor	Group 1 (Mean ± SD)	Group 2 (Mean ± SD)	t-value	p-value
Anticipation awareness	Age: 18–30 years vs 31–45 years	5.15 ± 0.70	5.18 ± 0.61	-0.37	0.711
	Field of study: health vs non-health	5.09 ± 0.69	5.26 ± 0.66	-2.08	0.038*
	Education: UG vs PG	5.09 ± 0.67	5.38 ± 0.70	-2.91	0.004*
	Residence: on-campus vs off-campus	5.16 ± 0.67	5.14 ± 0.70	0.24	0.808
Bothersome feelings	Age: 18–30 years vs 31–45 years	4.12 ± 0.71	4.19 ± 0.74	-0.66	0.509
	Field of study: health vs non-health	4.16 ± 0.72	4.08 ± 0.71	0.96	0.336
	Education: UG vs PG	4.07 ± 0.68	4.37 ± 0.83	-2.95	0.003*
	Residence: on-campus vs off-campus	4.07 ± 0.68	4.17 ± 0.74	-1.17	0.245
Cultural stigma beliefs	Age: 18–30 years vs 31–45 years	4.69 ± 0.81	4.56 ± 0.80	1.07	0.288
	Field of study: health vs non-health	4.53 ± 0.76	4.82 ± 0.82	-4.26	0.001*
	Education: UG vs PG	4.65 ± 0.79	4.75 ± 0.88	-0.83	0.408
	Residence: on-campus vs off-campus	4.61 ± 0.86	4.70 ± 0.77	-0.95	0.344
Debilitating attitudes	Age: 18–30 years vs 31–45 years	5.62 ± 0.80	5.63 ± 0.73	-0.04	0.969
	Field of study: health vs non-health	5.57 ± 0.75	5.72 ± 0.85	-1.53	0.127
	Education: UG vs PG	5.60 ± 0.79	5.73 ± 0.79	-1.17	0.244
	Residence: on-campus vs off-campus	5.58 ± 0.80	5.65 ± 0.78	-0.76	0.450
Natural perception	Age: 18–30 years vs 31–45 years	5.89 ± 0.57	5.80 ± 0.64	0.92	0.356
	Field of study: health vs non-health	5.86 ± 0.58	5.88 ± 0.59	-0.28	0.782
	Education: UG vs PG	5.87 ± 0.58	5.88 ± 0.59	-0.06	0.952
	Residence: on-campus vs off-campus	5.98 ± 0.62	5.81 ± 0.56	-2.45	0.015*
Empathy/supportive	Age: 18–30 years vs 31–45 years	5.81 ± 0.68	5.68 ± 0.74	1.25	0.213
	Field of study: health vs non-health	5.75 ± 0.72	5.86 ± 0.63	-1.28	0.200
	Education: UG vs PG	5.72 ± 0.70	6.06 ± 0.57	-3.45	0.001*
	Residence: on-campus vs off-campus	5.73 ± 0.70	5.82 ± 0.68	-1.08	0.282
Denial of effects	Age: 18–30 years vs 31–45 years	3.01 ± 0.78	3.11 ± 0.76	-0.86	0.392
	Field of study: health vs non-health	3.02 ± 0.81	3.03 ± 0.71	-0.10	0.918
	Education: UG vs PG	3.02 ± 0.79	3.02 ± 0.72	0.02	0.984
	Residence: on-campus vs off-campus	3.00 ± 0.80	3.04 ± 0.76	-0.35	0.724

According to Table 4, a multivariate analysis of variance (MANOVA) was performed to assess whether the combined menstrual attitude subscale scores differed significantly across sociodemographic factors (age, department, education level, and residence).

**Table 4:** MANOVA of sociodemographic factors on menstrual attitudes among male participants (n = 300) Multivariate analysis of variance (MANOVA) was conducted to assess the overall effect of sociodemographic variables on menstrual attitude subscales. Wilks’ lambda was used as the multivariate test statistic. A p-value < 0.05 was considered statistically significant.

Factor	Wilks’ Lambda	F-value	p-value
Intercept	0.017	2345.83	< 0.001
Age	0.984	0.67	0.702
Department	0.925	3.36	0.002
Education	0.913	3.95	< 0.001
Residence	0.970	1.29	0.255

Table 4 shows that there are statistically significant multivariate effects on the Menstrual Attitude Questionnaire - Indian Version (2024) subscales for both department (p = 0.002) and education level (p < 0.001). This suggests that participants' total menstrual attitude is influenced by their academic level (UG vs. PG) and field of study (health vs. non-health). The lack of significant effects (p > 0.05) for age and place of residence suggests that these factors may not have a major influence on the overall attitude dimensions. These results highlight the crucial role of disciplinary environment and educational experience in influencing male students' attitudes toward menstruation.

## Discussion

The current study provides insights into associations of menstruation attitudes among male Indian university students, adding significant understanding to a largely unexplored area of South Asian public health research. The study recorded attitudes on seven subscales, indicating a mix of supportive perceptions and persisting misconceptions, revealing enduring cultural myths and growing awareness.

The highest scores on the subscales measuring natural perception and empathy/supportive attitudes show that male students have become more aware that menstruation is a normal biological function that should be supported with empathy. These results are consistent with the findings of Gundi and Subramanyam (2020), who observed a combination of emotions in Indian adolescent boys: discomfort and humiliation along with interest and worry. This duality was primarily influenced by the lack of access to formal menstrual education [1]. Similar to Mason et al. (2017), whose qualitative investigation in India revealed that boys were willing to show sympathy and assist menstruating girls but were frequently constrained by cultural taboos and a lack of formal knowledge, the positive trend in empathy seen in this study supports their findings [6]. The importance of school-based health education in forming supportive male attitudes was highlighted by Chang et al. (2012), who found that adolescent males exposed to reproductive health content in Taiwan showed increased emotional engagement and knowledge [2].

The high mean score for Debilitating Attitudes (M = 5.57) indicates the persistence of ideas that women's menstruation physically weakens them and reduces their productivity, despite positive advances in natural perception. This is a reflection of deeper patriarchal narratives that regard menstruation as a sign of impurity or frailty, as discussed by Peranovic and Bentley (2017), who examined this cultural construct as a major obstacle to gender equity in India [3]. Remaining discomfort is further shown by the moderate scores on bothersome feelings and cultural stigma beliefs. Boys frequently absorb unfavorable opinions from friends, family members, and the media, particularly when menstruation is presented as shameful or taboo (Allen et al., 2011) [7]. Benshaul-Tolonen et al. (2020) illustrated how this stigma is expressed through social exclusion and teasing, particularly throughout adolescence, supporting the notion that unfavorable opinions regarding menstruation are context-dependent and socially learned [9].

The study discovered statistically significant correlations between disciplinary background and menstruation attitudes. Students from non-health departments showed lower natural perception scores and higher stigmatizing attitudes, indicating that those without formal health education might not know the truth and are more susceptible to cultural myths. This is consistent with the findings of Wong et al. (2013), who found that male health sciences students showed less stigma and more factual awareness than their business or law stream counterparts [13]. On the other hand, subscales measuring emotional sensitivity, empathy, and awareness showed consistently higher scores among postgraduate students. According to Fernández-Medina et al. (2023), who highlighted that young adults become more introspective about sexuality and social norms with age and education, this pattern demonstrates how educational maturity may be linked with students’ cognitive ability to critically engage with gendered issues [5]. The cross-sectional design precludes causal inference. The results further corroborate the construct validity of the Menstrual Attitude Questionnaire - Indian Version (2024) and are consistent with Roos (2018), who discovered that education level significantly predicted menstruation attitude scores among male students [14].

The natural perception scores of men living on campus were considerably higher than those of their off-campus counterparts. Campuses of universities frequently serve as microcultures that promote communication, diversity, and the availability of organized awareness campaigns. Additionally, students who live on campus might engage in casual peer talks or co-curricular sensitization initiatives more regularly. Social and cultural environments may play a critical role in shaping men's perceptions of women's reproductive health. Research suggests that individuals from conservative households or rural areas often possess limited awareness and hold more stigmatizing views regarding menstruation. These sociocultural influences can lead to persistent myths, embarrassment, and discriminatory behaviors toward menstruating individuals [8]. These variations demonstrate how crucial social learning contexts and formal curricula are in fostering gender-sensitive attitudes. Menstrual stigma may be lessened more naturally by informal learning through exposure to university events, workshops, or mixed-gender encounters than by textbooks alone.

The subscales Denial of Effects and Debilitating Attitudes also showed no significant differences across socio-demographic groups, which suggests that misconceptions about menstruation are firmly established across educational and social lines and may not be easily addressed through standard academic instruction. As noted by Rana et al. (2022), beliefs about menstruation are often passed down generationally and may persist even in well-educated populations unless directly challenged [11]. The fact that Denial of Effects scored the lowest (M = 3.04), however, suggests that most participants do not trivialize the menstrual experience, which is encouraging given that previous research has documented widespread beliefs among men that menstruation is exaggerated or irrelevant to them [11].

The study demonstrates that exposure and education are important factors in determining men's opinions regarding menstruation. Menstrual awareness must be incorporated immediately into non-health curricula intended for students studying other fields. Peer-led initiatives and coeducational conversations have been shown to be successful in lowering stigma and ought to be extended. Furthermore, our results support current demands for a menstruation-inclusive framework in health education that includes boys in the conversation around menstrual hygiene management (MHM). Both Allen et al. (2011) and Gundi and Subramanyam (2020) contend that attempts to destigmatize menstruation are insufficient if boys are not educated [1,7].

Menstrual suppression in high-performance settings, such as in the military (as reported by the US Army), shows how menstruation can impact diverse female populations and, by extension, the importance of male understanding in cohabited or professional environments [4].

Strengths

The study used the Menstrual Attitude Questionnaire-Indian Version (2024), a psychometrically tested and culturally appropriate instrument that ensures reliable measurement across important aspects of menstruation attitudes. An adequate number of participants (n = 300) from a variety of academic fields improves the findings' generalizability in a university setting. By including both undergraduate and graduate students, it is possible to examine educational attainment as a factor, providing deeper analytical insights. Using t-tests and MANOVA, the study offers a multidimensional analysis that allows for both univariate and multivariate interpretations of attitude differences.

Limitations

The study was restricted to university students, thereby excluding less-educated or nonacademic male populations whose attitudes may differ significantly. The ability to prove a causal relationship between menstruation attitudes and sociodemographic characteristics is limited by the cross-sectional methodology. Social desirability bias may have an impact on self-reported statistics, particularly when discussing delicate subjects like menstruation. The results may not accurately represent attitudes in rural or traditional societies because the study was carried out in institutional settings that were either urban or semi-urban.

## Conclusions

This study shows that Indian male university students' attitudes toward menstruation are nuanced and changing. Even while menstruation is becoming more understood and recognized as a normal and significant occurrence, several stigmatized myths and beliefs still exist, especially among undergraduates and non-health students. One significant influencing factor was found to be educational attainment. The results highlight the need to extend menstrual health education outside of the medical sector and into all other areas of higher education, especially for male students pursuing non-health fields who might not have regular access to reproductive health information. On-campus students scored higher in natural perception, demonstrating the importance of the residential context. The significance of the social and environmental milieu in forming gender-sensitive attitudes is highlighted by this research. Although these positive trends are encouraging, the consistency of some negative attitudes across sociodemographic groups, such as those measured by the Denial of Effects and Debilitating Attitudes subscales, indicates that some beliefs are ingrained and hard to overcome. This suggests that long-term, multifaceted interventions are required, involving community-based discussions, participatory learning, and cultural reinterpretation of menstruation as a common human concern, in addition to information transmission. This study shows that cultural conditioning and educational exposure both influence male attitudes; as a result, comprehensive, gender-inclusive policy and educational reforms are necessary for success. In the end, cooperation across educational institutions, families, health systems, and media outlets will be necessary to change menstruation from a subject of uncomfortable silence to one of knowledgeable empathy and supportive action that would empower male students to become advocates for menstrual health and dignity. It is a critical step toward dismantling stigma, promoting gender-sensitive health behavior, and ensuring the social and psychological well-being of all individuals affected by menstruation.
